# Understanding Australian Adolescents’ Perceptions of Healthy and Sustainable Diets, and Perceptions and Consumption of Pulses

**DOI:** 10.3390/nu18020265

**Published:** 2026-01-14

**Authors:** Adeline R. Lanham, Ayesha I. T. Tulloch, Jessica R. Bogard, Jolieke C. van der Pols

**Affiliations:** 1 Faculty of Health, School of Exercise and Nutrition Sciences, Queensland University of Technology (QUT), 149 Victoria Park Rd, Kelvin Grove, QLD 4059, Australia; j.vanderpols@qut.edu.au; 2 Centre for Agriculture and the Bioeconomy, Queensland University of Technology (QUT), 2 George St, Brisbane City, QLD 4001, Australia; ayesha.tulloch@qut.edu.au; 3 Centre for the Environment and Society, Queensland University of Technology (QUT), 2 George St, Brisbane City, QLD 4001, Australia; 4 Faculty of Science, School of Biology and Environmental Science, Queensland University of Technology (QUT), 2 George St, Brisbane City, QLD 4001, Australia; 5 Agriculture and Food, Commonwealth Scientific and Industrial Research Organisation (CSIRO), 306 Carmody Road, St Lucia, QLD 4067, Australia; jessica.bogard@csiro.au

**Keywords:** adolescents, pulses, sustainable diet, perceptions, consumption, Australia

## Abstract

**Background/Objectives**: To promote sustainable and healthy diets, increased consumption of pulses (the edible grains of legumes) is recommended. Adolescence is a period in which perceptions and behaviours develop that can impact lifelong dietary behaviours. Therefore, this study aimed to understand how Australian adolescents perceive healthy, sustainable diets and perceive and consume pulses. **Methods**: Students (11–17 years old, median = 15 years, n = 33) in Brisbane, Australia, participated in school-based face-to-face focus groups and an online survey. **Results**: Students’ perceptions of healthy and sustainable dietary behaviours largely focused on the health aspects of food and consideration of food waste. The main factors that adolescents identified as influencing the health and sustainability of their diet were a lack of perceived responsibility for the impact of their meal choices and lack of knowledge of what constitutes a healthy and sustainable diet. Most students were unfamiliar with the term ‘pulses’ and lacked a desire to consume pulses more often. Consumption of pulses was below recommendations in national and international dietary guidelines. The main factors that adolescents identified as influencing pulse consumption related to students’ lack of capability to prepare pulses and the limited opportunities to access pulse-based foods. **Conclusions**: Perceptions of sustainable diets and pulses were very limited, and a lack of knowledge, skills, and limited availability were identified as barriers to their consumption. Education and cooking classes, in conjunction with increased availability of tasty pulse foods, are recommended to increase students’ pulse consumption as part of a healthy and sustainable diet.

## 1. Introduction

Adolescence is a period of critical and formative development for attitudes and behaviours, as well as physical growth, yet research and strategies addressing poor nutrition among adolescents have been scarce in comparison to other age groups [1]. In a time when there is a need to shift to more healthy and sustainable diets [2,3], engaging with adolescents is an opportunity to educate and empower youths and support the formation of lifelong, positive dietary habits [1]. Institutions such as high-schools are recommended settings to implement strategies to support healthy and sustainable diets, as social and structural supports can drive behaviour change at the population level [3,4].

Increasingly, food-based nutrition guidelines are considering the environmental impacts of food production and consumption, due to ever-increasing impacts of agri-food systems on water availability, greenhouse gas emissions, biodiversity, and soil health [5]. Recommendations have consistently focused on diets rich in fruits, vegetables, and pulses, with animal products in moderation [6]. Adherence to dietary guidelines is low, with only 5% of Australians consuming the recommended intake of fruits and vegetables [7]. Diet quality is particularly poor amongst Australian adolescents’ and is characterised by overconsumption of processed foods and underconsumption of fruits and vegetables [8]. Recent data suggests that adolescents have a lower intake of legumes than any other age group (with the exception of 2–4 year olds) [9].

Increased consumption of pulses (edible dry seeds from legume plants including chickpeas, lentils, and dried beans) has been recommended to address the nutritional and environmental issues from current dietary patterns [3,10,11]. Pulses are low in energy and fat and high in complex carbohydrates, fibre, protein, and some trace elements including potassium, phosphorus, calcium, iron, and zinc [12]. Pulses also provide environmental benefits as they sequester nitrogen and phosphorus in the soil, reducing the need for synthetic fertilisers, and they increase water and land use efficiency in agricultural food production [13,14]. Their long shelf life and culinary versatility also contribute to the consumption of pulses worldwide [15]. However, consumption is below recommendations in many developed countries including Australia, with the average Australian consuming 12 g of legumes per day, short of the national guidelines of 150–300 g legumes per week and international guidelines of 75 g per day [9,16,17]. Pulse consumption is particularly low amongst adolescents [18,19].

To inform interventions aiming to promote the consumption of pulses by adolescents, it is crucial to understand the gap between dietary recommendations and consumers’ dietary habits and the factors influencing this [2]. The Behaviour Change Wheel (BCW) is a behaviour change framework that links factors influencing a behaviour with key intervention functions to support behaviour change [20]. Influencing factors have been categorised into three domains of behaviour (B) in the COM-B model: capability (C) (cognitive and physical capability), opportunity (O) (social and physical), and motivation (M) (automatic and reflective) [20]. The BCW and COM-B models have previously been used to review interventions to support sustainable diets [21], identify barriers and enablers to following dietary patterns [22,23], and inform interventions amongst adolescents [24].

Consumer perceptions, defined as ‘an idea, belief or image consumers have as a result of how they understand or see [foods]’ [25], play a key role in dietary behaviours [26]. A recent scoping review of evidence regarding adolescents’ perceptions of healthy and sustainable diets highlighted that, in general, adolescents are unsure about what constitutes healthy and sustainable eating, with particularly poor understanding of the environmental aspects [27]. However, the review indicated that this perception was influenced by the socioeconomic and cultural setting of the study population in some of the studies, suggesting the need for more data from more diverse settings about adolescents’ perceptions of healthy and sustainable diets and perceived barriers and facilitators of sustainable eating prior to designing a relevant intervention [27].

Amongst adult consumers of pulses, perceptions of pulses are largely positive [28,29]; however, perceptions of pulses amongst the broader population are not well understood, particularly amongst youth. In addition to lower consumption, research shows that younger populations have less knowledge of, and familiarity with, pulses [18,19,30]. Previously identified barriers to pulse consumption amongst adult consumers relate to capability and motivational domains of behaviour including lack of knowledge or skills to prepare and cook pulses, adverse gastrointestinal effects from pulse consumption, and a perceived misalignment with personal or social beliefs/norms (e.g., pulses perceived as for vegetarian or vegan consumers only) [29,31,32].

Australian adolescents’ perceptions of healthy and sustainable diets are unclear [27]. Furthermore, data reporting on Australian adolescents’ current pulse consumption and the factors influencing this are lacking. Addressing these gaps in the literature would enable the design of targeted strategies to improve pulse consumption. The aim of this study is to understand how Australian adolescents (1) perceive healthy, sustainable diets and (2) perceive and consume pulses, including drivers and enablers of consumption.

## 2. Materials and Methods

### 2.1. Study Design

A mixed-methods approach was taken to collect quantitative and qualitative data from a sample of high-school-aged adolescents in Australia. We conducted face-to-face focus groups and an online survey in a high school setting.

### 2.2. Participants and Recruitment

Participants were recruited from a public secondary school (i.e., a no-fee state high-school publicly funded and operated by the government) in a relatively mid to lower socioeconomic, metropolitan area of north Brisbane. The school was approached using convenience sampling through existing links between a research team member and a previous staff member of the school (there was no established relationship between the research team and the school or participants prior to study commencement). The high school had 1106 enrolled students across Years 7 to 12 (ages 11 to 18) at the time of the study. Research aims and methods were negotiated between the research team and school principal to ensure the project was practical within the school context and mutually beneficial.

Student leaders (voted by peers to represent their cohort) from Years 7–11 were invited to participate in an online survey and focus groups, resulting in a total of 33 participants. Student leaders were nominated for participation in this research by the school principal due to convenience and likelihood of attentiveness and engagement. Teachers shared consent forms and information sheets with invited students and their parents, after which parental consent for each participant was provided prior to the study commencing. Students’ informed consent was implied upon submission of the survey and attendance of the focus group, after having received written and verbal information about the project and given the opportunity to not participate. Information sheets intentionally described the purpose of the research as discussing ‘sustainable eating behaviours’, and not specifically ‘pulses’, to ensure participants’ knowledge and perceptions of pulses were not influenced by discussions or research stimulated by the information sheet provided [33]. Students were not formally compensated with payment for their time but were offered a pulse snack after participation.

### 2.3. Data Collection

Data were collected during lesson times at the end of the school year after all assessment had been completed to reduce academic disruption. The focus groups and online survey were undertaken in a quiet room in the school library with teacher supervision, and students were provided with computer access to complete the online survey. No identifiable data were collected from either the focus groups or online survey to protect participants’ privacy.

#### 2.3.1. Focus Group Data Collection

Semi-structured focus groups were conducted to facilitate discussion about students’ perceptions and consumption of sustainable diets and pulses. Students were divided into two age cohorts, (n = 13 from Years 7–9 (typically aged 11–14 years) and n = 20 from Years 10–11 (typically aged 14–16 years), and one focus group was facilitated with each age cohort separately to encourage participation and reduce potential bias in student responses caused by the perception of dominance due to age [34]. The number of participants in each focus group was greater than planned; therefore, rather than excluding participants, the focus group facilitator adapted the discussion structure to include smaller break-out groups to support all students’ participation. The groups were facilitated by AL who is a female dietitian and PhD candidate, with experience conducting focus group discussions for research. Each focus group ran for approximately 30 min and commenced with an introduction of the facilitator and reasons for the research, followed by an ‘icebreaker’ activity (each participant was encouraged to discuss their perceptions of a recently consumed, new food), three open-ended questions regarding perceptions and the importance of healthy and sustainable diets, and three open-ended questions regarding perceptions of and barriers and enablers towards consumption of pulses. Verbal examples of pulses and pulse-based foods were provided after participants shared their initial understanding of the terms ‘pulses’, ‘legumes’, and ‘dried beans and peas’. The questions were structured to address the components in the COM-B model [35]. Participants were then offered an opportunity to add anything else to the discussion (see facilitation guide in Supplementary File S1). Discussions were audio-recorded, transcribed verbatim using QUT’s secure audio transcription platform (QUT Transcribe, Amazon Web Services, Sydney, Australia), then cleaned for errors. The facilitator also took written notes of relevant non-verbal communication. All data were collected in English.

The focus group schedule was piloted with a group of four adolescents known to the research team who had parental consent to participate. Following piloting, minor adjustments were made to the schedule and questions, to ensure students were supported to discuss with each other rather than with the facilitator in an interview style.

#### 2.3.2. Online Survey Data Collection

A structured survey (Supplementary File S2) was developed to collect data from adolescents about their perceptions and consumption of pulses. Quantitative data about the frequency, type, and form of pulses consumed, and food preparation and purchasing behaviours, were collected from individual students and collated using the survey platform Qualtrics [36]. Demographic data including the students age, sex, residential postcode (as a proxy for socioeconomic status), language spoken at home (as a proxy for cultural background), and diet identity (e.g., vegetarian, vegan, etc.) were also collected.

The survey comprised 14 questions, including dichotomous, multiple choice, check multiple box selection, 5- and 7-point Likert scale matrices ranging from “never” to “everyday”, and free text open-response-style questions. The survey focused on current pulse products available in Australia [15] and was adapted from a previous survey [29].

The survey was piloted with three adolescents known to the research team prior to the main data collection to ensure that the survey language was appropriate, easy to use, and expected duration of completion was accurate (10 min).

### 2.4. Data Analysis

#### 2.4.1. Focus Group Data Analysis

Qualitative data from focus groups was transcribed, cleaned, and analysed as per Braun and Clarke (2012) [37]. Data were coded inductively using a Microsoft Excel spreadsheet by one author. Thematic analysis was performed, with codes grouped into themes deduced from the COM-B model [20]. The identification of themes and supporting codes was undertaken by one author, then reviewed and agreed upon by two other authors of the research team. Some codes were identified as supporting more than one component of the COM-B model and therefore categorised under more than one theme but discussed under that which the authors agreed was most appropriate. Suggested interventions were derived directly from participants’ responses and categorised according to the BCW.

#### 2.4.2. Online Survey Data Analysis

Data from the survey was collated and cleaned using Qualtrics (Qualtrics XM, 2024). Survey quality was checked for completeness, duration of completion, unusual patterns of responses to multiple choice or matrix questions, and appropriateness of open text responses [38]. Demographic characteristics (age, sex, socioeconomic indexes for areas score of residential suburb (derived from residential postcode), language spoken at home, and diet identity) were analysed by count and percentage using Qualtrics StatsIQ [36]. Regular pulse consumers were defined as consuming at least one type of pulse containing food a minimum of some (two to three) days per week, or consuming two or more types of pulses at least once per week, as per the Australian Dietary Guidelines [16]. Descriptive statistics were reported according to regular pulse consumption, and statistical tests to predict group differences between regular pulse consumers and non-regular pulse consumers were performed using Fisher’s Exact Test for categorical variables and *t*-test for age.

### 2.5. Ethical Approval

Ethical approval for this study was provided by the QUT Human Research Ethics Committee (project 5879, approved 20 December 2022), and the project was registered with the Queensland Education Research Inventory (project number 550/27/2672).

## 3. Results

### 3.1. Participant Characteristics

A total of 33 participants were involved in the focus groups, and 32 participants completed the online survey, of which eight (25%) were identified as regular pulse consumers (Supplementary Table S1). Participants were predominantly female (64%) with a median age of 15 years (range = 11–17 years). Participants were aged 11–15 years in the first focus group (FG1) (median = 14 years) (n = 13) and 14–17 years in the second focus group (FG2) (median = 16) (n = 20). Three participants (9%) reported an Aboriginal or Torres Strait Islander background. All participants spoke English with eight students (25%) additionally speaking another language at home. There were no statistical differences between regular pulse consumers and non-regular pulse consumers in age, sex, socioeconomic status, indigenous status or language spoken at home.

Most participants (n = 24, 73%) consumed diets with no restrictions; however, two participants reported following a vegetarian diet, and a further six participants excluded other foods (gluten, dairy, nuts, and fish). The main drivers of usual dietary patterns were family norms (mentioned by n = 25 (78%) overall; n = 5 (62%) among regular pulse consumers), followed by taste/texture preferences (mentioned by n = 17 (53%) overall; n = 3 (38%) among regular pulse consumers), and health/nutrition (mentioned by n = 14 (44%) overall; n = 3 (38%) among regular pulse consumers). Most (83%) of the food exclusions reported were due to known allergies/intolerances, with three (38%) regular pulse consumers reporting a food allergy/intolerance. The two vegetarian students were non-regular pulse consumers, and reported following a vegetarian diet due to animal welfare concerns (n = 2, 100%) or for health and environmental concerns (n = 1, 50%). Impact of the environment was considered by only three (9%) participants overall, two of which were regular pulse consumers (25%).

### 3.2. Focus Group Results—Perceptions and Consumption of Healthy and Sustainable Diets

Healthy and sustainable dietary behaviours received 155 mentions, mostly related to consumption of un/healthy food items (n = 133), with less mentions of healthy dietary patterns and habits (n = 14) and sustainable dietary behaviours (n = 8). Identified themes are listed in Table 1 and key quotes in Supplementary File S3. Across the COM-B model, most mentions related to opportunity or motivation.

#### 3.2.1. Capability

Adolescents rarely spoke about their perceived capability to make healthy and sustainable dietary choices and made no mention of the physical skills involved in healthy and sustainable dietary behaviours. There appeared to be a lack of ability to discriminate a healthy diet from a sustainable diet—one participant asked, “*Was it not the same?*”(FG1).

#### 3.2.2. Opportunity

The environmental opportunities mentioned to consume a healthy and sustainable diet were predominantly related to the physical food system (n = 33, 21%). Adolescents discussed sustainable food production involving no pesticides, living off the land, prioritising diversity of plants cultivated, consideration of the ecosystem, and consumption of locally grown foods. Purchasing environments associated with healthy and sustainable diets were local fruit shops rather than large supermarkets. However, students reported that accessibility and convenience of the food environment also influenced purchasing behaviours.

Waste was the most frequently discussed aspect of the food system, with 17 individual mentions. This included minimising waste (using a shopping trolley rather than plastic bags to transfer food items), prioritise recyclable and biodegradable materials used to package or serve foods (cans, bottles, paper, and cardboard), using reusable materials such as cloth grocery bags, sorting and composting waste, and reducing potential food waste resulting from the aesthetic standards of fresh produce at supermarkets. One participant stated *“we see a lot of fruit and vegetables that go to waste, though, because they don’t look presentable. Like if we started to eat more of those, I think it would be helpful”* (FG2).

A broader global perspective of the food system was mentioned by older adolescents, with comparison of the Australian waste management system to other developed countries-*“I don’t think we do enough in Australia in terms of like when you go to Europe and things like that. They’re recycling glasses, plastics, everything… And here none of that is recycled. You know the plastics are, but that’s about it”* (FG2).

The types and accessibility of foods available were also discussed. Adolescents mentioned changing the types of protein rich foods to consume “*less red meat*”(FG2) and insects –“*they do it in Asia…Why don’t we? … Have you eaten an insect before? They are not bad! Grasshoppers are actually good!”* (FG2).

Other factors impacting consumption of healthy and sustainable diets were cost and time. Older adolescents mentioned that their own income enables more frequent use of the school tuckshop [school canteen], as they prioritised sleep in the morning over preparing their own lunch.

In terms of social influences, some older adolescents mentioned the cultural norms in Australia contributing to a lack of care for the environment. In the context of comparing European and Australian attitudes towards the environment and responsible disposal of waste, one adolescent stated *“…whereas in Australia it’s just, yeah, who gives a toss?”* (FG2).

#### 3.2.3. Motivation

A large portion of the discussion centred on reflective motivation to consume healthy and sustainable diets (n = 48, 31%). The importance or value of healthy eating was mentioned 17 times and described as *“good”* or *“important”* (FG1 and FG2). The value of sustainability of diets or the environment was mentioned less frequently (n = 11 mentions) and often with less certainty, for example, *“pretty important”* (FG1). However, when asked to consider whether the environmental or health impact of their diet was more important, both were considered important (n = 2 valued either health or the environment more highly, n = 3 valued both equally).

Within statements regarding the value of the environment and healthy and sustainable diets, competing priorities of school, time, sleep, and money were mentioned as barriers to engaging in such behaviours-*“I feel like [environmental sustainability] is important but it’s not our top priority because of school. We’re putting all of our assessments first”* (FG2).

Positive consequences of healthy and sustainable diets were mentioned including those relating to health and nutrition and future generations.

The benefit to sports performance was also mentioned by several participants-*“I reckon if we had a good diet it would be pretty beneficial because we are active… for our performance and achievements”* (FG2). However, sport was also mentioned as facilitating consumption of more unhealthy foods—*“I think as long as you’re eating in moderation with what you’re doing, so like you can have some unhealthy foods, let’s say, you know, you go for a run or you go to the gym or you play sports, you can have that little bit of unhealthy food…”* (FG2).

The role and responsibility of individuals and the community in consuming healthy and sustainable diets was not discussed directly. However, participants did mention their individual capacity to choose what types of foods they consume (e.g., blemished or oversized fresh produce) and from where (locally sourced). Adolescents expressed being primarily responsible for their own behaviours and limited effort in changing peers’ behaviour.

There were several mentions of emotions contributing to healthy and sustainable dietary behaviours. Several older participants mentioned stress as an inhibitor to making healthy and sustainable dietary choices—*“I mean, [eating sustainably] is very important, and it’s like a pressing issue that I think we all worry about, but we’re all just too stressed to do anything.. We’re just stressed about everything*” (FG2).

One participant expressed disappointment at others’ lack of care for the environment; however, other participants expressed ambivalence towards healthy and sustainable diets-*“I think most of us agree that we don’t really care what we eat”* (FG2).

### 3.3. Focus Groups Discussion Results—Perceptions and Consumption of Pulses

From the focus group data, there were 224 discussion points from participants in the context of consuming pulses which sat across all sources of behaviour from the COM-B model. Identified themes are listed in Table 2 and key quotes in Supplementary File S4.

#### 3.3.1. Capability

Participants frequently made statements regarding their perceived capability to prepare and consume pulses (n = 93). The psychological capability of adolescents to consume pulses was mentioned most often in relation to their knowledge of pulses (or lack thereof) or ability to acquire more information. When asked what the adolescent participants thought of with regards to pulses, most responses indicated a lack of knowledge (n = 20), with 14 of these responses indicating confusion or that they *“don’t know”* or have *“no idea”* what pulses or legumes are. Two responses associated pulses with “*throbbing*” (pulsating), and one with ‘impulse’ buying.

Whilst some participants were able to correctly state examples of pulses or pulse dishes (beans (n = 4), lentils (n = 1), kidney beans (n = 1), chickpeas (n = 1), the school tuckshop burrito bowl (n = 1), chilli con carne (n = 1), and hummus (multiple, with prompting)), these were often posed as a question requiring confirmation, for example, *“Is that like beans and stuff?”* (FG1). One respondent was able to state an example of a pulse, but with some confusion regarding categorisation in the Australian Guide to Healthy Eating [36] *“I remember it was like, somewhere near like, with milk or something, but I’m not sure where, but, I remember it would be lentils, definitely”* (FG1). Others were able to identify that pulses are commonly used as a protein source in vegetarian or vegan dishes-*“Aren’t they, like, substituted for meat? Like vegetarian and vegan options?”* (FG2)

In addition to participants’ current knowledge, they also discussed how to acquire information about pulses. Some potential sources of information mentioned were from other pulse consumers in their social networks (*“Maybe if you just know that someone who regularly ate them, you’d ask them”* (FG2)), the internet (Google, videos, and YouTube), cooking shows, cookbooks, instructions on product labels, or home economics classes.

The physical capability to prepare and cook pulses was the most common theme emerging from the focus group (n = 38). Most mentions of cooking pulses referred to adolescents acquiring the skills to cook pulses for themselves, rather than their current skills (or lack of)—*“Even, like, you know how you can do cooking at school? If you just, even, you incorporate that into, like, “right, today we’re cooking this dish”, and you know, it’s something that not a lot of kids have had before, so they learn how to make it”* (FG2).

Participants also referred to the value of practicing learned cooking skills and how this may promote longer-term consumption of pulses than acquiring pre-made meals-*“Once you learn, it’s like if you make it and you want to make it, and you keep making it, like, it’s stuck in your brain. Like, it becomes more of what you do”* (FG2).

When asked whether participants would prefer support to have the capability to prepare pulses for themselves or opportunities to access ready-made pulse-rich foods, from both focus group discussions, six stated a preference for learning to cook, four stated access to prepared pulse dishes, and three stated both.

#### 3.3.2. Opportunity

When asked about social influences on their pulse consumption, adolescents identified people that they know whom regularly consume pulses and parents and teachers as sources of relevant information. The cultural norms of pulse consumption were mentioned by one older adolescent.

*“If you look at the foods we eat in Australia, they sort of, like, stem from cultures. Like, we’ll have a lot of pasta, the Italian and, you know, things like that. But where they eat a lot of lentils and things like that, that’s not really like a widespread meal option, or thought about. Like, I don’t eat a lot of them. I have eaten them before, but I reckon if you go outside and ask 20 people if they’ve eaten lentils before, at least 18 will say no.”*

(FG2)

The social influence of peers was also discussed as to whether they would support pulse consumption, with mixed responses from ambivalent (*“I don’t think [my friends] would notice”* (FG1)) to positive (*“They might want to try it”* (FG1)).

Discussions of physical opportunities to consume pulses largely centred on the availability of pulse foods for consumption. The most frequently mentioned way for pulses to be consumed was in main dishes (n = 22) such as soups, Mexican foods (chilli con carne or a burrito bowl from the tuckshop), curry, and baked beans. Mentions of dishes were commonly stated alongside familiarity and taste. Suggestions of opportunities to consume more pulses included as an addition to familiar meals (whole or in the form of a powder or paste) (n = 6) or in ultra-processed (n = 2) or snack foods (n = 1), such as commercial chickpea burgers—*“If I choose to eat them I’d get one of those chickpea burgers.”* (FG2)

Schools were discussed as a potential environment to increase familiarity with pulses or their consumption through the tuckshop (n = 12), home economics classes (n = 8) or the broader school curriculum (n = 2). Some participants suggested that offering pulse foods through the tuckshop would be more effective than teaching adolescents to cook pulse foods for themselves, due to the convenience (“S*ometimes you are very busy with school and you just don’t feel like cooking*” (FG1)) and their current limited responsibility for food preparation.

Adolescents agreed that consumption of pulse foods at home would be facilitated by learning to cook dishes with which they were familiar at school.

*“Because you like, eat [a prepared pulse dish from the school tuckshop] and touch it, like, but you don’t know how to make it. Like, when you’re out of school, how are you going to eat it?”*

(FG1)

One participant mentioned the potential approaches of exposing school students to production aspects of the food system, as well as preparation and consumption of pulses.

*“if we have vegetable patches or, like, even if you just get agriculture, it’s like, it’s offered at most schools where, you know, you learn how to grow different types of vegetables, different types of fruits. And then obviously you can incorporate that into your home ec’ classes, then you don’t have to go buy it. You’ve got that natural stuff there. And then you can teach people how to, you know, cook it as well, so then you kind of full circle. You’re teaching people how to grow it, teaching people how to cook it, and then people are eating.”*

(FG2)

Within the food environment, packaging and accessibility of pulses was mentioned in the context of making pulse purchasing appealing and easy.

*“And I know like you can buy chickpeas and things like that, like canned, but like they’re often, like, I don’t know, like the Aldi I go to, they’re in the very back corner like sort of hidden away from the rest of the vegetables.”*

(FG2)

Time and effort to cook and consume pulses was mentioned several times as a barrier: (*“If you’re actually cooking your food, it will take more planning, maybe a meal plan of what you’re going to cook which day? What you need to buy…”* (FG2)). Cost was referred to both as a barrier (*“and sometimes it can be more expensive”* (FG2)) and facilitator of pulse consumption (*“…But it would also be cheaper to [cook] it yourself…”* (FG2)).

#### 3.3.3. Motivation

Motivational factors that are automatic and internal were frequently mentioned by adolescents as a factor in pulse consumption, specifically familiarity of pulse foods (n = 22), taste (n = 13), stress (n = 2), satiety (n = 1), and visual appeal (n = 1). Most participants mentioned consuming pulses in familiar dishes would be beneficial—*“Probably incorporating them in dishes that you already know that you like?”* (FG2). However, one participant suggested trying new dishes when introducing pulses—*“I think it would be better with new foods. Because if you don’t like it with that, there’s a chance that you don’t really like the food all up*.” (FG1)

Most previous eating experiences of pulses that were discussed by participants implied negative perceptions of taste. Pulses were described as *“gross”* (FG2) and unsatiating. To overcome the poor taste, suggestions were made to mask the taste with other flavours and make the foods look appealing.

*“For example, like, mushrooms-noone likes mushrooms. Except for [XX]. So… my mum used to, like, hide them, not hide them, but, like, put in a dish where you can’t really taste them. You can taste, like, other flavours that taste way better… like sauce or something.”*

(FG1)

Two adolescents also mentioned stress as a barrier to consuming pulses, due to the time required to prepare them.

Participants consciously considered the desirability of or intent to consume pulses. Multiple adolescents declined wishing to consume more pulses, with few stating positive intentions of pulse consumption.

*“So I think it’s about just knowing your intention, saying “Alright, I like them. I’m gonna go cook them.” So I think if you introduce [pulse dishes in home economics] more to the youth and maybe they’ll cook it and eat it. And then, yeah, start eating it more, yeah.”*

(FG2)

Adolescents also discussed the convenience of preparing and acquiring pulse dishes as another motivating factor hindering pulse consumption (n = 8). This related to the time required, as well as the accessibility, to prepare or purchase pulse food. One adolescent mentioned that their role in food preparation was limited, as their parents prepared most meals. They suggested that, due to this, they had little motivation to learn to cook pulse dishes for themselves.

*“It also depends on age, like, if you’re an adult and you’re living independently by yourself, you go to that time to prepare yourself so it would be more beneficial to know how to prepare them. But as teenagers we kind of rely on what our parents cook for us and make for us every day. So, like, and for the young people would be just convenience rather than learning how to prepare them.”*

(FG2)

### 3.4. Pulse Survey Results

The online survey had a response rate of 97% (n = 32). There was one incomplete survey which was excluded from analysis. The mean duration of time to complete the survey was three minutes (range = 1.5–10 min).

#### 3.4.1. Food Preparation of Adolescents

**Figure 1:**
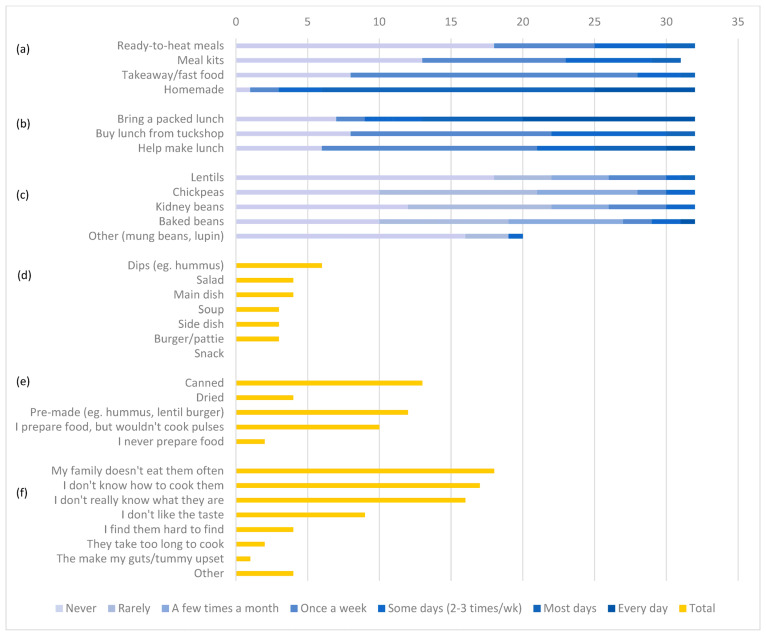
Type and frequency of dinner meal consumed (n = 32) (**a**), lunch prepared (n = 32) (**b**), and pulses consumed (n = 32) (**c**) by Australian adolescent participants; number of Australian adolescent participants reporting pulses consumed by dish (n = 6) (**d**), forms of pulses used in food preparation (n = 26) (**e**), and barriers to pulse consumption (n = 32) (**f**).

The results from the survey indicated that for dinner, the majority of students consumed homemade foods most days of the week; however, most students have takeaway/fast food at least once a week (75%) (Figure 1a). Meal kits and ready-to-heat meals were consumed by students, but only approximately one quarter of adolescents reported their regular consumption (more than twice per week).

In terms of school lunches, 60% of students bring a packed lunch on most or every day of the week, and most participants reported being involved in preparing their own lunch (81%). Use of the tuckshop was also common, with 75% of students using the tuckshop at least once a week (Figure 1b).

#### 3.4.2. Adolescents’ Pulse Consumption

Only six participants (18%) reported consuming any type of pulse regularly (at least 2–3 days per week). Four participants (12%) reported never consuming any type of pulse, with 11 (34%) participants only reporting consumption of baked beans. Statistical tests indicated no significant differences in characteristics between regular pulse consumers and non-regular pulse consumers and as such are not discussed in detail here (Supplementary Table S1).

The most commonly consumed type of pulse reported by students was baked beans, with 40% of students consuming baked beans at least a few times a month (Figure 1c). For the students that consumed pulses regularly (n = 6), dips were the most commonly consumed pulse product (n = 6), followed by in a salad (n = 4) or main dish (e.g., curry, lentil bolognese) (n = 4). No participants reported consuming pulses as a snack (e.g., roast chickpeas) (n = 0) (Figure 1d).

Fourteen students reported cooking with pulses, and of these, 13 (93%) used canned pulses, 12 (86%) used pre-made processed pulse-based foods, and 4 (29%) used dried pulses (Figure 1e). A further ten students (71%) are involved in food preparation but do not cook with pulses, and two participants (14%) never prepare food.

The most frequently reported barriers to pulse consumption were related to usual family eating habits (n = 18, 56%) and a lack of knowledge of pulses (n = 16, 50%) or how to cook them (n = 17, 53%) (Figure 1f). Other barriers reported included unpleasant taste or texture, low availability or accessibility, time required to prepare, and gastrointestinal symptoms.

## 4. Discussion

This study sought to identify Australian high-school students’ perceptions of healthy and sustainable diets and their perceptions and consumption of pulses. We found that adolescents recognise the need to make healthy, sustainable diet choices, but these choices are constrained by opportunity, capability, and motivation. Our results support previous findings that most adolescents are unaware of what constitutes both a healthy and sustainable diet, with greater knowledge of the health aspects of their diet than how their dietary choices affect the environment [27]. Furthermore, most adolescents were unfamiliar with the term ‘pulses’ (when used to imply grain legumes) and lack motivation to consume more pulses. Our data indicated that the frequency of pulse consumption is lower than the national and international dietary guidelines [3,16]. This study found that the main factors that influence pulse consumption relate to students’ lack of skills and knowledge to prepare pulses, lack of cultural norm to consume pulses, and reduced accessibility and availability of pulse foods at their home or school.

These findings indicate that improved education about the impact of the food system and foods consumed on people’s livelihoods, health, and the environment is needed to address adolescents’ common knowledge deficit and inattention to the consequences of their dietary choices [39,40]. Our study revealed some misunderstandings, for example, about available recycling options for food packaging, which could be addressed through improved education on this topic. Student awareness of the Australian Guide to Healthy Eating can be used as partial evidence of effective nutrition education, which is part of the school curriculum from the earliest year of schooling [41]. However, despite the environmental sustainability of food choices similarly included in the national curriculum from the earliest school year [41], adolescents were unable to clearly identify sustainable or both healthy and sustainable dietary behaviours. This indicates a need to equip teaching staff with the knowledge and capacity to deliver relevant teaching material, such as through home economics classes [42]. Previous effective education interventions targeting adolescents about the impacts of dietary choices have used creative or practical teaching techniques [43]. However, there is limited evidence to support the effectiveness of education as an isolated strategy on a change in dietary behaviour [44].

In addition to educational strategies, environments in which adolescents engage with food can be restructured to support access to convenient and appealing healthy and sustainable food choices, including pulses [45]. High schools have been recommended as an opportune setting to implement changes to food environments due to the potential to facilitate behaviour change on a larger, community scale than individual-focussed interventions and build on the existing institutional structure of schools [46]. Australian adolescents have reported increasing the availability of appealing, healthy choices as a preferred strategy to improve eating behaviours [47] and are largely motivated by direct and tangible consequences of their dietary choices (primarily taste, as well as enjoyment, cost, and convenience) [45,48]. Opportunities suggested from this study include access to compost and recycling bins, locally grown fruit and vegetables, convenient, affordable, and appealing plant-based foods (including pulses), and reducing and recycling food packaging. Previous studies have also simultaneously incorporated strategies such as persuasion and incentivisation through persuasive labelling, advertising highlighting the positive taste, health or environmental attributes, and eye-catching placement to promote the selection of healthy and sustainable food options [49,50]. When implemented in schools, the broader evidence indicates that these strategies are most effective when tailored to a specific population and co-designed with adolescents [27,50]. Importantly, the primary model for sourcing and preparing meals in the context of the target population must be considered as this can vary greatly (e.g., lunchbox model with or without a tuckshop (as common in Australia and in this study’s sample population), full meal provision, or outsourcing meals from home or local food outlets), influencing the autonomy of adolescents’ food choice, and should guide the relevant strategies that may be implemented and be considered in the identification of stakeholders.

Adolescents’ frequent mentions of the need for skills and knowledge suggest a need for practical training for the identification, selection, preparation, and consumption of pulses [20]. In an Australian study, adults also reported a lack of knowledge and skills as a barrier to consuming pulses [29], which suggests that most parents or caregivers may not be confident in teaching adolescent children how to cook pulses or cooking pulses at home themselves. This is likely to contribute to the major barrier to consuming pulses among adolescents as identified in this study, which is that pulses were not offered by their parents as part of meals consumed at home. Most adolescents in this study indicated that they participate in preparing their own lunch and therefore would have the opportunity to directly apply learned pulse preparation skills to their own food. Theoretical and practical teaching through high school home economics classes have been mentioned by adolescents as preferred ways to learn about food preparation, as mentioned in this study and elsewhere [47]. Home economics classes are known to positively impact food and nutrition knowledge and skills, self-efficacy, and dietary behaviours [51,52,53]; however, home economics classes are only offered in some Australian high schools, at each school’s discretion [42]. Other methods of increasing knowledge and skills to prepare pulses by adolescents include using engaging mediums such as videos, recipe books, and face-to-face cooking demonstrations [54].

From this study, Australian adolescents’ consumption of pulses was low, with only 18% of adolescents meeting the national recommendations of consuming two serves of pulses per week [16]. This is lower than the proportion of Australian adults who meet this recommendation (28%) [55]. Adolescents in this study consumed processed pulses (baked beans) more often than minimally processed pulses that are often incorporated into vegetable-rich meals (kidney beans, chickpeas, and lentils). Although this was not reported in this study, this may be due to the palatability and convenience of baked beans. However, study participants who were regular consumers of pulses (>2–3 times/week) were more likely to consume hummus (homemade or commercial) or pulses in a main meal, rather than in a processed form. The convenience of preparing and consuming hummus, cultural norms to include pulses in household meals, and more readily accepted taste of pulses by regular consumers may explain why these two forms of pulses were most commonly consumed regularly [29]. Pulses play a more dominant role in some food cultures than others [56]. While 25% of the study participants spoke a language other than English at home, there was no indication during the focus groups that students considered their family’s food culture when discussing their perception of pulses, although we did not specifically elicit this information. Future studies might consider using targeted questioning to better reveal whether cultural backgrounds play a part in pulse consumption [56].

Easily prepared foods using canned pulses (such as hummus, chickpea salad, Mexican beans, and lentil soups) may be more likely to be adopted and normalised than dishes using dried pulses that require more time and resources for soaking and cooking, particularly if they are to be prepared by adolescents themselves, with adequate knowledge and skills. Canned pulses can be cheap and quicker alternative to dried pulses, thereby being more likely to be adopted into regular family meals [57]. Despite having a similar nutritional content (especially when drained and rinsed) [57], due to imports and offshore canning, canned pulses are likely to have a greater impact on the environment than dried pulses [58]. However, both canned or dried pulses have a much lower environmental impact in comparison to the animal-sourced protein foods which they might replace [59]. Traditional main dishes that include pulses and other vegetables such as Indian dahl, Mexican chilli con carne or French soups provide nutritional benefits from the other vegetables in the dish and may be appealing and familiar to Australian consumers due to the cosmopolitan foodscape. Recipes and practical tips to facilitate simple and tasty inclusion of canned pulses in household meals are recommended to support regular consumption of pulses at home [60].

In this study, the collection and analysis of both quantitative and qualitative questionnaire data and qualitative data from semi-structured focus groups enabled deep insight into Australian adolescents’ opinions and experiences regarding healthy and sustainable diets and pulse consumption. However, this study was not without limitations. This study was conducted with student leaders attending a government-operated high school in a metropolitan area of Australia characterised by easy access to a variety of fast food outlets and mid to lower socioeconomic advantage; therefore, findings may have varied should the study have been conducted in schools with different socioeconomic, cultural, and geographical characteristics, or even amongst the wider school population within the same sample school. Although the participants were selected to be representative of the school students, to maximise appropriate participation, all participants were ‘student leaders’ and therefore likely to exhibit a higher level of engagement than some of their peers; however, there are no indications that their perceptions of sustainable diets and pulses are much different from their peers. Perceptions and consumption of pulses and sustainable diets among adolescents may be different among those from other socioeconomic or geographic areas within Australia due to access, familiarity, and exposure to pulses. Pulses are most commonly consumed in African, Asian, and South American cuisines—particularly in Indian, Turkish, and Middle Eastern dishes; therefore, familiarity and consumption of pulses are likely to be strongly influenced by cultural background [61]. Surveys were completed immediately following the focus groups sessions; therefore, discussions with peers and the focus group facilitator may have impacted the survey results. Similarly, as focus groups with each age cohort were conducted on consecutive days, it is possible that discussions with younger participants from FG1 may have influenced data collected from older participants in FG2. The sample size and amount of data collected from the focus groups was limited by school and student availability, rather than data collection ceasing at the point of data saturation, as is common in qualitative studies [37], which may have limited the variety and depth of themes identified. The research team considered the sample size and resulting data to be sufficient in order to guide future interventions for the school, whilst preserving a collaborative relationship with the school staff. Quantification of dietary pulse intake was measured by self-reported short questions regarding frequency of consumption only, not by a systematic total dietary assessment method; thus, the extent to which dietary guidelines for pulses were met may be subject to over- or under-reporting. Future studies investigating adolescents’ perceptions of healthy and sustainable diets, and pulses, and quantitative consumption data of pulses should be undertaken with different socioeconomic and cultural groups and larger sample sizes to provide more generalisable results for the Australian adolescent population and guide future scalable interventions. In the preliminary stages of this study, several challenges were experienced in the ethical approval processes and recruitment of schools to conduct research across multiple jurisdictions and locations, which had been the original goal. Therefore future studies with Australian adolescents should develop and nurture relationship with schools and plan the research resources needed for broader, more representative data collection efforts. Alternatively, future research could consider alternate settings such as recreational or sporting facilities, or digital platforms for data collection or delivery of interventions; however, sample bias must be considered in these settings. Tailoring interventions to a specific target population and their key food environments, food knowledge and attitudes, and eating patterns will help ensure that strategies are effective at addressing different population needs [27].

## 5. Conclusions

Australian adolescents’ knowledge of healthy and sustainable diets and pulses is poor, and consumption of pulses is below the recommended amounts. However, motivation towards improving the sustainability of adolescents’ diets and increased consumption of pulses was expressed, and major factors influencing their capability and opportunity to consume a healthy and sustainable diet and pulses were identified, including a lack of knowledge and skills, lack of cultural norms, and reduced accessibility and availability of pulse foods.

This study makes it clear that education, practical training (including cooking classes), and changes to the food environment are needed to inform and empower adolescents to make food choices that are healthy and sustainable and to support adolescents to increase their consumption of pulses. Schools provide a key opportunity to implement these strategies, with the institutional structure to enable the implementation of policies to encourage and monitor change in the food provision and marketing at schools and relevant curriculum such as home economics [10,60]. To ensure strategies are effective, it is recommended that interventions are multicomponent (not education alone), tailored to the specific population, sustainable over the long term, and co-designed with key stakeholders, including adolescents, to meet their needs and preferences [27].

## Figures and Tables

**Table 1:** Factors identified influencing adolescents’ perceptions and consumption of healthy and sustainable diets, and suggested interventions, based on the Behaviour Change Wheel [20].

Behaviour					
Consumption of healthy foods/limited consumption of unhealthy foods (n = 133)					
Healthy dietary consumption pattern (n = 14)					
Sustainable behaviours (n = 8)					
**Capability (n = 1)**		**Opportunity (n = 45)**		**Motivation (n = 59)**	
***Psychological***	***Physical***	***Social***	***Physical***	***Reflective***	***Automatic***
Lack of knowledge (n = 1)		Cultural norms (n = 2)	School tuckshop (n = 6)	Value of healthy eating (n = 17)	Stress (n = 3)
			Global food system (n = 4)	Value of sustainable diets (n = 6)	Ambivalence (n = 3)
			Food production and soil impacts (n = 7)	Value of the environment (n = 5)	Disappointment (n = 1)
			Transport and packaging (n = 3)	Benefit for sports performance (n = 5)	
			Food accessibility (n = 1)	Energy balance of unhealthy foods (n = 2)	
			Food environment (n = 1)	Health and nourishment (n = 4)	
			Waste (n = 17)	Competing priorities (n = 4)	
			Time (n = 1)	Consumer role and responsibility (n = 2)	
			Cost (n = 1)	Future generations (n = 1)	
			Alternative protein sources (n = 2)	Convenience (n = 1)	
**Suggested interventions**					
Education in school curriculum regarding what constitutes a health and sustainable diet, and the role of consumers					
Restructuring of food environment to improve access and appeal of healthy and sustainable foods, including attractive labelling and advertising					

**Table 2:** Factors identified influencing adolescents’ perceptions and consumption of pulses, and suggested interventions, based on the Behaviour Change Wheel [20].

Behaviour					
Consumption of pulses (n = 224)					
**Capability (n = 93)**		**Opportunity (n = 79)**		**Motivation (n = 55)**	
***Psychological***	***Physical***	***Social***	***Physical***	***Reflective***	***Automatic***
Lack of knowledge (n = 20)	Cooking and food preparation skills (n = 38)	Parents’ influence (n = 3)	Pulses in main dishes (n = 22)	Convenience (n = 8)	Familiarity (n = 22)
Knowledge (n = 17)	Practice (n = 1)	Peers’ influence (n = 2)	School tuckshop (n = 12)	Desirability (n = 3)	Taste (n = 13)
Media as an information source (n = 9)		Teachers’ influence (n = 2)	Home economics classes (n = 8)	Intention to consume pulses (n = 3)	Stress (n = 2)
Social networks as an information source (n = 7)		Cultural norms (n = 1)	Pulses as a meal addition (n = 6)	Lack of desirability of foods (n = 1)	Appealing foods (n = 1)
Food labelling as an information source (n = 1)		Pulse consumers (n = 1)	Time (n = 6)	Role in food preparation (n = 1)	Lack of satiety (n = 1)
			Pulses in the food environment and accessibility (n = 3)		
			Cost (n = 3)		
			Pulses in ultra-processed foods (n = 2)		
			School curriculum (n = 2)		
			Pulse production (n = 1)		
			Pulse transport and packaging (n = 1)		
			Pulses as snacks (n = 1)		
**Suggested interventions**					
Education regarding the benefits and ways of consuming pulses					
Restructuring of food environments to increase the access and appeal of pulse foods, including attractive labelling and advertising					
Training adolescents to equip them with the skills to prepare tasty and/or familiar pulse foods for themselves					

## Data Availability

The raw data supporting the conclusions of this article will be made available by the authors on request.

## Supplementary Materials

The following supporting information can be downloaded at: https://www.mdpi.com/article/10.3390/nu18020265/s1, Table S1: Socioeconomic and cultural characteristics of adolescent participants; File S1: Focus group schedule; File S2: Online survey questions; File S3: Thematic summary of perceptions of healthy and sustainable diets; File S4: Thematic summary of perceptions of pulses.

## Abbreviations

The following abbreviations are used in this manuscript:

BCW	Behaviour Change Wheel
COM-B	Capability, Opportunity, and Motivation for Behaviour
FG	Focus Group
