# Supplementary material for: Understanding Australian Adolescents’ Perceptions of Healthy and Sustainable Diets, and Perceptions and Consumption of Pulses
Source: Nutrients. 2026 Jan 14;18(2):265. doi: 10.3390/nu18020265 (PMC12845408; doi:10.3390/nu18020265)
Supplement: Supplementary file 1 [file nutrients-18-00265-s001.zip › Supplementary Table S1.pdf]

Supplementary Table 1: Socioeconomic and Cultural Characteristics of Adolescent Participants

Participants	Total (n=32 (100%))	Regular pulse consumer (n=8 (25%))	Non-regular pulse consumer (n=24 (75%))	p-value
Age (mean, years)	15.0 (SE=1.6)	14.9 (SE=0.55)	15.0 (SE=0.34)	0.90[1]
Gender				0.40[2]
Male	10 (33%)	1 (13%)	9 (38%)	
Female	21 (64%)	7 (88%)	14 (58%)	
Prefer not to say	1 (3%)	0 (0%)	1 (4%)	
Area index of disadvantage (quintiles)*				0.31[3]
First quintile (most disadvantaged)	2 (6%)	1 (13%)	1 (4%)	
Second quintile	0 (0%)	0 (0%)	0 (0%)	
Third quintile	27 (84%)	7 (88%)	20 (83%)	
Fourth quintile	3 (9%)	0 (0%)	3 (13%)	

Fifth quintile (least disadvantaged)	0 (0%)	0 (0%)	0 (0%)	
Aboriginal or Torres Strait Islander status				0.55[4]
Aboriginal	2 (6%)	0 (0%)	2 (8%)	
Aboriginal and Torres Strait Islander	1 (3%)	0 (0%)	1 (4%)	
Neither	29 (91%)	8 (100%)	21 (88%)	
Language(s) spoken at home				0.64[5]
English	31 (97%)	8 (100%)	23 (96%)	
Other (Urdu, Telugu, Tagalog, Danish, Samoan, Cantonese, Spanish)	8 (25%)	1 (13%)	7 (29%)	

*derived from Socio-Economic Indexes for Areas (SEIFA) ranks areas according to relative socioeconomic disadvantage from postcode of residence, quintiles

[1]t-test comparing mean age of regular pulse consumers and non-regular pulse consumers

[2]Fisher's Exact test comparing proportion of male and females, between regular pulse consumers and non-regular pulse consumers

[3]Fisher’s Exact test comparing quintile of residential area of disadvantage, and regular pulse consumers and non-regular pulse consumers

[4]Fisher’s Exact test comparing proportion of those identifying as indigenous or non-indigenous, between regular pulse consumers and non-regular pulse consumers

[5]Fisher’s Exact test comparing proportion of those speaking English only or another language at home, between regular pulse consumers and non-regular pulse consumers
