# Supplementary material for: Understanding Australian Adolescents’ Perceptions of Healthy and Sustainable Diets, and Perceptions and Consumption of Pulses
Source: Nutrients. 2026 Jan 14;18(2):265. doi: 10.3390/nu18020265 (PMC12845408; doi:10.3390/nu18020265)
Supplement: Supplementary file 1 [file nutrients-18-00265-s001.zip › File S1- Focus group schedule.pdf]

File S1: Focus group schedule

(5 mins) Introduction of researchers and purpose of research and today's focus group:

- Researchers name and role
- Purpose of research
- Confirm consent to participate
- Confirm consent for audio-recording. Remind students to please not use names of people or the school during the recorded session

(5mins) Icebreaker: Discussion of favourite foods

(5mins) Q1. How important is healthy eating to you?

(5mins) Q2. How important is environmental sustainability to you?

(5mins) Q3. What do you think a 'healthy and sustainable' way of eating might look like?

(10mins) Q4. What first comes to mind when you think about 'pulses'?

Prompt: What about 'legumes'?

(10mins) Q5. Would you like to eat more pulses?

Why?

Why not?

What might your friends think?

(10mins) Q6. If you wanted to eat more pulses, how would you do this?

Where would you go?

Feel like you know how to prepare?

Do you have the resources that you need – money, cooking equip etc?

Feel like you have the skills?

(5mins) Q7. Is there anything else you would like to add to what we have discussed today about pulses?

Conclusion and thanks

(Time in brackets are suggested limits)
