# Supplementary material for: Understanding Australian Adolescents’ Perceptions of Healthy and Sustainable Diets, and Perceptions and Consumption of Pulses
Source: Nutrients. 2026 Jan 14;18(2):265. doi: 10.3390/nu18020265 (PMC12845408; doi:10.3390/nu18020265)
Supplement: Supplementary file 1 [file nutrients-18-00265-s001.zip › File S2 - Online survey questions.pdf]

File S2: Online Survey questions

What is your age?

- 11 years
- 12 years
- 13 years
- 14 years
- 15 years
- 16 years
- 17 years
- 18 years

What is your gender?

- Boy
- Girl
- Non-binary
- Prefer not to say

What is the postcode of where you normally live?

- *Open*

We are interested in how people's background may influence the foods they eat. **How would you describe your family's background?** (you may choose up to two (2) options):

- Indigenous Australian
- Australian (excluding Indigenous Australians)
- New Zealand or Pacific Islander
- British (eg. from England)
- European (eg. German, Italian)
- Asian
- American
- African
- Middle Eastern
- Other:
- Unsure
- Prefer not to say

We are interested in usual eating patterns or restrictions. **Which of the following options best describes how you usually eat?**

- No restrictions (eat most foods including meat and fish)
- Vegetarian (avoid eating meat or fish, but eat eggs and dairy)
- Vegan (avoid eating all animal products - ie. avoid meat, fish, eggs and dairy)
- Other:

What are your main reasons for eating this way?

(please rank up to three (3) reasons)

- My family eats this way
- The taste and textures of these foods
- Health and nutrition
- Environmental concerns
- Animal welfare concerns
- Allergies/intolerances
- Other:

Most weeks, how often do you...?

	Never	Rarely	Some days (2-3 times/wk)	Most days	Every day
Help make lunch or dinner	-	-	-	-	-
Bring a packed lunch to school	-	-	-	-	-
Buy lunch from the school tuckshop	-	-	-	-	-

For dinner, what kind of food do you usually eat?

- Home made (from scratch)
- Meal kits (eg. HelloFresh, taco kits, curry packs etc)
- Ready-to-heat meals (eg. frozen meals, Lite n' Easy, microwave meals)
- Take away/fast food

Have you heard of these terms?

	Yes	No
Pulses		
Legumes		
Dried beans and peas		

Here are some examples of pulses:

- Chickpeas – commonly used in hummous, salads or stews

- Kidney beans – commonly used in dishes such as Mexican chilli beans or soups
- Lentils – commonly used in dahl or soup
- Baked beans – commonly used to made baked beans
- Other beans such as edamame, white beans, cannellini beans, mung beans, pigeon peas, broad beans, lupin

How often would you eat each type of pulse?

	Never	Rarely	A few times a month	Once a week	Some days (2-3 times a week)	Most days (4-5 times/wk)	Every day
Chickpea	-	-	-	-	-	-	-
Lentils	-	-	-	-	-	-	-
Baked beans	-	-	-	-	-	-	-
Kidney beans	-	-	-	-	-	-	-
Other:	-	-	-	-	-	-	-

How do you usually eat pulses? (you may choose more than one option)

- Soup (eg. minestrone soup)
- Salad (eg. chickpea or lentil salad)
- Snack (eg. roast chickpeas)
- Side dish
- Main dish (eg. curry, lentil bolognese)
- Burger/pattie (eg. lentil pattie)
- Dip (eg. hummus)
- Other:

If you were making a dish with pulses, what type would you use? (you may choose more than one option) *(only an option if involved in making of lunch and dinner)*

- Dried
- Canned
- Pre-made form (eg. hummus, lentil burger etc)
- I prepare food, but wouldn't choose to prepare pulses
- Other:

Why don't you eat pulses? (you may choose more than one options) (*Only an option if never eating pulses)*

- I don’t know what they are
- My family never eats them
- I don’t like the taste
- I don’t know how to cook them
- They make my gut/tummy upset
- Other:
