# Supplementary material for: Understanding Australian Adolescents’ Perceptions of Healthy and Sustainable Diets, and Perceptions and Consumption of Pulses
Source: Nutrients. 2026 Jan 14;18(2):265. doi: 10.3390/nu18020265 (PMC12845408; doi:10.3390/nu18020265)
Supplement: Supplementary file 1 [file nutrients-18-00265-s001.zip › File S3- Thematic summary HSD.pdf]

File S3: Key quotes from focus groups regarding healthy and sustainable diets

Codes	Key quote/s
Capability	
Lack of knowledge	*"Was it not the same?" (FG1, F)*
Opportunity	
Cultural norms	*"It's just a cultural thing, we seem to like neglect our environment as long as long as everyone's making money that seems to be alright." (FG2, M)*<br>*"...whereas in Australia it's just, yeah, who gives a toss?" (FG2, M)*
School tuckshop	*"I think convenience as well is something that contributes to how our diet is. Like the tuckshop, there's food there. I'd go to the tuckshop everyday, just find that it's easy. And I don't have to wake up early and make lunch." (FG2, M)*
Global food system	*"I don't think we do enough in Australia in terms of like when you go to Europe and things like that. They're recycling glasses, plastics, everything… And here none of that is recycled. You know the plastics are, but that's about it." (FG2, M)*<br>*"Yeah, I think it yeah, like it applies pressure to other nations, like [Speaker 2] was saying, Europe's all close. So if you do something in one country, it impacts the other country. So now there's pressure on everyone to do better…" (FG2, M)*
Food production and soil impacts	*"I was thinking about where it's produced, no matter how it's produced so that if it's grown where it degrades the soil then it means that it impacts future farming, like, how it grows back, or other plantations and things like that. Or if it it's a grown in a way that depletes the soil-" (FG1, F)*<br>*"Living off the land." (FG2, M)*<br>*"No pesticides." (FG2, M)*
Transport and packaging	*"Locally and sustainably sourced." (FG 2, F)*<br>*"Less packaging as well like plastic and everything." (FG2, F)*<br>*"So, packaging, paper or yeah, like paper bags. I feel like that's more sustainable than plastic 'cause it's biodegradable and you can regrow more trees so it's pretty sustainable" (FG2, M)*
Food accessibility	*"Like the tuckshop, there's food there. I'd go to the tuckshop every day, just find that it's easy." (FG2, M)*
Food environment	*"Don't shop at Woolies. Shop at, like, a fruit shop." (FG2, F)*
Waste	*"We see a lot of fruit and vegetables that go to waste, though, because they don't look presentable. Like if we started to eat more of those, I think it would be helpful." (FG2, F)*
Time	*"I'd go to the tuckshop everyday, just find that it's easy. And I don't have to wake up early and make lunch." (FG2, M)*
Cost	*"The ones that have jobs [use the tuckshop every day]." (FG2, M)*
Alternative protein sources	*"Less red meat." (FG2, M)*
	"Insects...they do it in Asia…Why don't we? … Have you eaten an insect before? They are not bad! Grasshoppers are actually good!" (FG2, M)
Motivation	
Value of healthy eating	*"I think [healthy eating] is important." (FG1, F)*
Value of sustainable diets	*"Pretty important." (FG1, F)*
Value of the environment	*"I think it's important. I like going for hikes and things like that. So I don't like when I see, like, um, you're a few k's out in the middle of nowhere and there's just rubbish lying around. It's disappointing." (FG2, M)*

	“I mean, it's very important, and it's like a pressing issue that I think we all worry about, but we're all just too stressed to do anything.” (FG2, F)
Benefit for sports performance	*"[Healthy eating is] good for sport." (FG1, M)*<br>*“I reckon if we had a good diet it would be pretty beneficial because we are active… for our performance and achievements." (FG2, M)*
Energy balance of unhealthy foods	*“So long as I'm exercising, I don't really care what I eat.” (FG1, M)*<br>*“I think as long as you're eating in moderation with what you're doing, so like you can have some unhealthy foods, let's say, you know, you go for a run or you go to the gym or you play sports, you can have that little bit of unhealthy food.” (FG2, M)*
Health and nourishment	*“[Healthy eating] gives your body nutrients and stuff.” (FG1, F)*
	“[Healthy eating] prevents you from being sick.” (FG1, F)
Competing priorities	*“As long as long as everyone's making money that seems to be alright.” (FG2, M)*
Consumer role and responsibility	*“We see like a lot of fruit and vegetables that go to waste, though, because they don't look presentable. Like if we started to eat more of those, I think it would be helpful.” (FG2, F)*
Future generations	*“[Sustainable eating is important when] thinking of the future [generations].” (FG1, F)*
Convenience	*“I think convenience, as well, is something that contributes to how our diet is.” (FG2, M)*
Stress	*“[Sustainable eating] is very important, and it's like a pressing issue that I think we all worry about, but we're all just too stressed to do anything.” (FG2, F)*
Ambivalence	*“No, I think most of us agree that we don’t really care what we eat.” (FG2, F)*<br><br>*“I don't really take notice of my diet. Um, so long as I'm exercising, I don't really care what I eat. Umm but my diet's pretty standard. It's breakfast, lunch and dinner. Nothing special about it.” (FG2, M)*
Disappointment	*“I don't like when I see, like, um, you’re a few k’s out in the middle of nowhere and there's just rubbish lying around. It's disappointing.” (FG2, M)*
