# Supplementary material for: Understanding Australian Adolescents’ Perceptions of Healthy and Sustainable Diets, and Perceptions and Consumption of Pulses
Source: Nutrients. 2026 Jan 14;18(2):265. doi: 10.3390/nu18020265 (PMC12845408; doi:10.3390/nu18020265)
Supplement: Supplementary file 1 [file nutrients-18-00265-s001.zip › File S4 - Thematic summary Pulses.pdf]

File S4: Key quotes from focus groups regarding pulses

Codes	Key Quotes
Capability	
Lack of knowledge	*"I have no idea" (Multiple)*<br>*"I don't know what that is" (FG1, M)*<br>*"What is a pulse?" (FG1, M)*<br>*"I still don't know what a legume is." (FG1, M)*<br>*"I didn't know there were chickpeas in hummus" (FG1, M)*<br>*"I think of pulses, like blood pulses" (FG2, F)*<br>*"Like 'impulses'? Like when you go shopping hungry and you buy more than what you need." (FG2, M)*
Knowledge	*"Is that like beans and stuff?" (FG1, M)*<br>*"Wasn't lentil that, like.. legumes, aren't they lentils?" (FG1, F)*<br>*"What about red kidney beans? Do they count?" (FG1, M)*<br>*"Like chickpeas?" (FG2, F)*<br>*"Aren't they like substituted for meat? Like vegetarian and vegan options?" (FG2, M)*<br>*"Protein? Don't you have to eat like a lot, though? To be able to match how much…-" (FG2, F)*
Media as an information source	*"Watch a cooking show." (FG1, F)*<br>*"Look it up. Might search for a video or something." (FG1, F)*<br>*"Google a professional chef". (FG1, F)*<br>*"Just search it up online" (FG1, M)*
Social networks as an information source	*"Maybe if you just knew that someone who regularly ate them, you'd ask them" (FG2, F)*<br>*"Ask your parents [how to prepare pulses]" (FG1, F)*
Food labelling as an information source	*"[Look for information on] the instructions on a baked beans can or something?" (FG1, M)*
Cooking and food preparation skills	*"Even, like, you know how you can do cooking at school? If you just, even, you incorporate that into, like, "right, today we're cooking this dish", and you know, it's something that not a lot of kids have had before, so they learn how to make it" (FG2, M).*<br>*"[It would be helpful] if you knew how to cook [pulses]." (FG1, F)*<br>*"Because you, like, eat [pulse foods at the tuckshop] and touch it like, but you don't know how to make it. Like when you're out of school, how are you going to eat it?" (FG1, F)*
Practice	*"I reckon yeah, because once you learn, it's like if you make it and you want to make it and you keep making it like, it's stuck in your brain, like, it becomes more of what you do." (FG2, F)*
Opportunity	
Parents' influence	*"[Ask] my Dad [how to cook pulses]" (FG2, M)*<br>*"As teenagers we kind of rely on what our parents cook for us and make for us every day" (FG2, M)*
Peers' influence	*"I don't think [peers] would have a problem with [me eating more pulses]" (FG1, F)*<br>*"[Peers] might want to try [eating pulses]" (FG1, F)*
Teachers' influence	*"[Ask] a teacher [how to cook pulses]" (FG1, M and F)*
Cultural norms	*"If you look at the foods we eat in Australia, they sort of like stem from cultures. Like we'll have a lot of pasta, the Italian and you know, things like that. But where they eat a lot of lentils and things like that, that's not really like a widespread meal option, or thought about like I don't eat a lot of them. I have eaten them before, but I reckon if you go outside and I ask 20 people if they've eaten lentils before, at least 18 will say no. So..-" (FG2, M)*

Pulse consumers	*"Maybe if you just knew that someone who regularly ate them, you'd ask them [how to cook pulses]" (FG2, F)*
Pulses in main dishes	*"My mum puts them in soup" (FG1, F)*<br>*"It depends on what we are cooking….. Sometimes if we are making a curry or we might put them in soups.." (FG2, M)*<br>*"Well, they may not be really nice on their own, but if you, like, take the time and cook them into a dish. Like lentil soups and things like that, then they're actually alright." (FG2, M)*
School tuckshop	*"Aren't they in, like, the burrito bowl in the tuckshop?"(FG1, F)*<br>*"Now I feel like once you finish school, like if you're having a burrito bowl at school from the tuck shop, when you finish school or when you get home, maybe you wanna, like, learn how to make it yourself so you can have it." (FG2, M)*<br>*"You, like, eat [pulses dishes from the tuckshop] and touch it" (FG2, F)*
Home economics classes	*"Even, like you know how you can do cooking at school? If you just even you incorporate that into, like "right, today we're cooking this dish," and you know it's something that not a lot of kids have had before, so they learn how to make it. And then obviously subsequently you get to try it. And if they enjoy it, then that might, mean more people might do it." (FG2, M)*<br>*"With our home ec' things that we used to have in grade seven and 8 and 9, I remember that like when you had in home ec' classes but if they even had it in those, like, I probably would like, probably wanna make it. There were, like, some things that we made that I wanted to make at home a lot. So, like, I, I love having fried rice. It was like the one thing that I always made for a while." (FG2, F)*<br>*"If we have vegetable patches or like even if you just get agriculture, it's like, it's offered at most schools where, you know, you learn how to grow different types of vegetables, different types of fruits. And then obviously you can incorporate that into your home ec' classes that you don't have to go buy it. You've got that natural stuff there. And then you can teach people how to, you know, cook it as well, so then you kind of full circle. You're teaching people how to grow it, teaching people how to cook it, and then people are eating." (FG2, M)*
Pulses as a meal addition	*"My mum used to like hide [mushrooms]... put in a dish where you can't really taste them. You can taste like other flavours that taste way better.... like sauce or something." (FG1, F*<br>*"And when they're in dishes that that, like, make them taste better. Like, not, like on its own or something like that." (FG1, F)*<br>*"[It would be helpful to] probably incorporate them in dishes that you already know that you like?" (FG2, F)*<br>*"Learning how to make dishes with [pulses] at school… Like home economics" (FG1, F)*
Time	*"Could take the time to cook a decent dish and make them taste better." (FG2, M)*<br>*"I think [time and stress] is why people don't do [cook with pulses more]" (FG2, F)*<br>*"It would definitely take more planning. Like, if you're, if you're actually cooking your food, it will take more planning, maybe a meal plan of what you're going to cook which day? What you need to buy…" (FG2, M)*
Pulses in the food environment and accessibility	*"And I know like you can buy chickpeas and things like that, like canned, but like they're often like I don't know, like the Aldi I go to, they're in the very back corner like sort of hidden away from the rest of the vegetables." (FG2, M)*
Cost	*"Maybe you wanna, like, learn how to make it yourself so you can have [the burrito bowl from the tuckshop]. But it would also be cheaper to do it yourself… "[FG2, F)*<br>*"Sometimes it can be more expensive [to cook at home]" (FG1, F)*<br>*"You can incorporate [the pulses grown in agriculture classes] into your home ec' classes that you don't have to go buy it." (FG2, M)*<br>*"…But it would also be cheaper to [cook] it yourself…" (FG2, M)*

Pulses in ultra-processed foods	"If I choose to eat them I’d get one of those chickpea burgers" (FG2, M)<br>"If feel like they were in, like fast food, well not fast food, but those burgers…[mumbling] if they were in there…[mumbling] even if you’d never had it, I’d probably, like, try them." (FG2, F)
School curriculum	"They are really hard to grow… We had to do a thing [with pulses] in grade six…" (FG1, F)
Pulse production	“…if we have vegetable patches or, like, even if you just get agriculture, it's like, it’s offered at most schools where, you know, you learn how to grow different types of vegetables, different types of fruits. And then obviously you can incorporate that into your home ec’ classes, then you don't have to go buy it. You've got that natural stuff there. And then you can teach people how to, you know, cook it as well, so then you kind of full circle. You're teaching people how to grow it, teaching people how to cook it, and then people are eating.” (FG2, M)
Pulse transport and packaging	“Making the packaging like, look more appetising.” (FG1, F)
Pulses as snacks	"Sometimes you are very busy with school and you just don’t feel like cooking so having them like snacks and easy to make things might make it more-" (FG1, F)
Motivation	
Convenience	"[It would be important for pulse dishes to be] readily available because people are very lazy." (FG2, M)<br>“Sometimes you are very busy with school and you just don’t feel like cooking” (FG1, F)
Desirability	"I could really go for some baked beans right now….I wanna go home so I can eat some baked beans. I’m just really craving them right now." (FG2, F)<br>"I'd be open to eating more [pulses]" (FG2, M)
Intention to consume pulses	“So I think it's about just knowing your intention, saying “Alright, I like them. I'm gonna go cook them.”” (FG2, M)
Lack of desirability of foods	"Even if a pea touches my plate, I’m going to starve" (FG1, F)
Role in food preparation	“It also depends on age like if you're adult and you’re living independently by yourself. You go to that time to prepare yourself so it would be more beneficial to know how to prepare them, but as teenagers we kind of rely on what our parents cook for us and make for us every day. So like, and for the young people would be just convenience rather than learning how to prepare them.” (FG2, M)
Familiarity	"If you're having a burrito bowl at school from the tuck shop, when you finish school or when you get home, maybe you wanna, like, learn how to make it yourself so you can have it." (FG2, m)<br>"You know how you can do cooking at school? If you just even you incorporate that into, like “right, today we're cooking this dish,” and you know it's something that not a lot of kids have had before, so they learn how to make it. And then obviously subsequently you get to try it. And if they enjoy it, then that might, mean more people might do it." (FG2, M)
	“Probably incorporating them in dishes that you already know that you like?” (FG2, F)
Taste	"And when they're in dishes that that, like, make them taste better." (FG1, F)<br>"[Pulses] are gross" (FG2, F)<br>"I don't like the taste [of pulses]" (FG2, F)<br>"Could take the time to cook a decent dish and make them taste better.<br>“Like, I'm just saying, like, if, like, for example, like, mushrooms - noone likes mushrooms. Except for [XX]. So, like, my mum used to like hide them, not hide them, but, like, put in a dish where you can't really taste them. You can taste like other flavours that taste way better.... like sauce or something.” (FG1, F)<br>“I'd be open to eating more...Well, they may not be really nice on their own, but if you, like, take the time and cook them into a dish. Like lentil soups and things like that, then they're actually alright.” (FG2, M)

Stress	*"I think [time and stress] why people don't [cook with pulses more]" (FG2, F)*
Appealing foods	*“Making the packaging, like, look more appetising.” (FG1, F)*
Lack of satiety	*“[Pulses] are gross. Doesn’t really fill you up either.” (FG2, F)*
